# Vision-Based UAV Detection and Localization to Indoor Positioning System

**DOI:** 10.3390/s24134121

**Published:** 2024-06-25

**Authors:** Kheireddine Choutri, Mohand Lagha, Souham Meshoul, Hadil Shaiba, Akram Chegrani, Mohamed Yahiaoui

**Affiliations:** 1 Aeronautical Sciences Laboratory, Aeronautical and Spatial Studies Institute, Blida 1 University, Blida 0900, Algeria; lmohand@yahoo.fr (M.L.); akramchegrani11@gmail.com (A.C.);; 2 Department of Information Technology, College of Computer and Information Sciences, Princess Nourah Bint Abdulrahman University, P.O. Box 84428, Riyadh 11671, Saudi Arabia; sbmeshoul@pnu.edu.sa; 3 Department of Computer Science, College of Computer and Information Sciences, Princess Nourah Bint Abdulrahman University, P.O. Box 84428, Riyadh 11671, Saudi Arabia

**Keywords:** unmanned aerial vehicles, indoor positioning system, computer vision, stereo vision, visual odometry, depth estimation, triangulation

## Abstract

In recent years, the technological landscape has undergone a profound metamorphosis catalyzed by the widespread integration of drones across diverse sectors. Essential to the drone manufacturing process is comprehensive testing, typically conducted in controlled laboratory settings to uphold safety and privacy standards. However, a formidable challenge emerges due to the inherent limitations of GPS signals within indoor environments, posing a threat to the accuracy of drone positioning. This limitation not only jeopardizes testing validity but also introduces instability and inaccuracies, compromising the assessment of drone performance. Given the pivotal role of precise GPS-derived data in drone autopilots, addressing this indoor-based GPS constraint is imperative to ensure the reliability and resilience of unmanned aerial vehicles (UAVs). This paper delves into the implementation of an Indoor Positioning System (IPS) leveraging computer vision. The proposed system endeavors to detect and localize UAVs within indoor environments through an enhanced vision-based triangulation approach. A comparative analysis with alternative positioning methodologies is undertaken to ascertain the efficacy of the proposed system. The results obtained showcase the efficiency and precision of the designed system in detecting and localizing various types of UAVs, underscoring its potential to advance the field of indoor drone navigation and testing.

## 1. Introduction

In modern society, Unmanned Aerial Vehicles (UAVs) have become versatile aviation systems with a broad range of applications. Initially developed for military use, these drones are highly efficient tools capable of performing various tasks at a low cost and without risk in sectors such as agriculture [1], search and rescue [2] and disaster prevention [3]. UAVs can be controlled using various technologies, including GPS, cameras, remote control, and even speech [4]. For instance, GPS can determine position and altitude, cameras can capture images not visible to the naked eye, and laser sensors can measure distances between objects. This collected data can then be utilized for autonomous flight or object recognition.

Indoor positioning systems (IPS) are technologies designed to locate objects or people within indoor environments where GPS signals are often weak or unavailable. These systems are increasingly important for applications such as navigation within large buildings, asset tracking, and enhancing user experiences in smart buildings. However, indoor positioning techniques face several challenges [5]. The complexity of indoor environments, with obstacles like walls and furniture, can cause signal interference and multipath effects, reducing accuracy. Additionally, the diversity of building materials and layouts adds to the difficulty of creating a universally reliable system. The need for high precision, real-time updates, and cost-effectiveness further complicates the development and deployment of effective indoor positioning solutions [6].

Indoor drone detection and positioning methods are game-changers for a variety of reasons. On the safety and security front, accurately monitoring these interior flyers avoids collisions with people, objects, and other drones. Real-time position data also enable the surveillance of unauthorized drone activities, which is particularly critical in sensitive areas such as government buildings, data centers, and high-security institutions. Accurate drone placement also improves navigation and control significantly, which is critical for enabling autonomous operations. These methods encompass Ultrawideband (UWB) [7], Ultrasonic (US) positioning using sound waves [8], Radio-Frequency Identification (RFID) for asset tracking [9], Visual Recognition (VR) via cameras and computer vision [10], Acoustic Positioning (AP) with microphones and sound analysis [11] and sensor fusion for improved accuracy [12]. The author in [5] presents a survey of IPS methods and technologies by focusing on the research cited in prior surveys, providing a unique perspective that assesses the current status of the field based on existing survey literature. In [13], a comparison of IPS technologies based on their cost, accuracy, performance, and complexity is given. Hybrid methods are being explored as a means to address the constraints associated with individual IPS technologies.

This paper explores the implementation of IPS structure utilizing advanced computer vision technology. The proposed system aims to detect and accurately locate UAVs within indoor environments through an enhanced vision-based triangulation method. Compared to other IPS technologies, this approach offers significant advantages, including higher precision in complex environments, improved reliability in the presence of obstacles, and reduced dependency on external signals such as Wi-Fi or Bluetooth. By leveraging computer vision, the system can achieve superior accuracy and robustness, making it an ideal solution for a wide range of indoor applications. The structure of the remainder of this paper is as follows: Section 2 discusses the related works in the field of IPS. Section 3 presents the hardware and materials employed for configuring the system. In Section 4, details of the proposed method for UAV detection, including dataset preparation and model training, are provided. The discussion on depth measurement is provided in Section 5, starting with a description of depth estimation methods, followed by the application of triangulation for position determination, and concluding with the enhancement of estimation stability using a complementary filter. Section 6 describes the position estimation in the proposed method. Section 7 conducts an evaluation of the system’s performance and analyzes the results obtained. Lastly, Section 8 presents conclusions drawn from the study and outlines potential avenues for future research.

## 2. Related Works

Unmanned Aerial Vehicles (UAVs), based on outdoor positioning technology, have found extensive applications in military, industrial, agricultural, entertainment, and other domains. However, their reliance on outdoor GPS signals poses challenges when used indoors or in environments with weak or no GPS signal. This limitation restricts the potential applications of UAVs that rely on indoor positioning technology. In recent years, there has been a growing industry demand for UAV inspection systems that utilize indoor positioning technology, prompting increased focus on issues such as control optimization, path tracking, and related challenges in this context. A review of indoor positioning systems for UAV localization using different machine learning algorithms can be found in [14]. In [15], the development of a UAV indoor positioning system using ArUco fiducial markers is presented, while [16] aims at assessing the positioning accuracy of UWB-based positioning thanks to the comparison with positions provided by a motion capture (MoCap) system. Authors of [17] propose a technique to enhance the precision of UWB by integrating visual-inertial odometry (VIO) positioning technology. In [18], a design approach for indoor positioning, which combines the Inertial Measurement Unit (IMU) with UWB positioning technology based on an unscented Kalman filter, is introduced. This approach effectively mitigates IMU error accumulation, leading to a substantial enhancement in positioning accuracy. Otherwise, achieving reliable pose determination often demands substantial computational resources. To address this issue, ref. [19] introduces an innovative real-time visual compass for estimating the three Degree-of-Freedom (DoF) relative orientations of an RGB-D camera, incorporating the Surface-Normals-Based RANdom Sample Consensus Model (RANSAC). Moreover, ref. [20] introduces a UAV localization algorithm based on deep neural networks. Additionally, genetic algorithms (GA) are used to identify the optimal set of hyperparameters for the deep neural network. In [21] particular attention was devoted to the potential of utilizing light signals as a promising and dependable solution to the indoor positioning challenge. The authors of [22] discuss the limitations of Bluetooth-based fingerprinting and IMU-based localization by introducing an innovative system that leverages IMU sensors and BLE beacons based on a Trusted K nearest Bayesian Estimation (TKBE) algorithm. Because it is challenging to estimate the parameters of the path loss model using conventional techniques, ref. [23] suggests utilizing the Particle Swarm Optimization (PSO) method to simulate the parameter estimation process. While traditional proposals employ only one network technology, ref. [24] integrates two different technologies (self and remote positioning) with a multimodal fingerprint-based approach in order to provide improved accuracy.

Although numerous technologies are under investigation, an accurate and dependable indoor positioning system has not yet surfaced. Lately, cost-effective and versatile visual sensors have emerged as advantageous tools for UAV navigation due to the swift progress in computer vision. Key aspects of visual navigation include visual localization and mapping, obstacle avoidance, and path planning. A thorough examination of techniques for UAV navigation based on vision is conducted in [25]. The existing methods are systematically categorized and thoroughly evaluated based on their capabilities and characteristics. Visual odometry (VO) and visual inertial odometry (VIO) represent a category of vision-based navigation that assesses the robot’s movement (both rotation and translation) and determines its location within the surroundings. Current cutting-edge techniques for VO and VIO are surveyed in [26]. In outdoor settings with strong GNSS satellite signals, UAVs can attain consistent positioning and navigation. Nonetheless, GNSS signals become susceptible to disruption and may be inaccessible in environments where GNSS reception is obstructed, like indoor or underground areas. For this purpose, the authors of [27] propose a monocular VIO with point-line feature fusion and adaptive nonlinear optimization in the backend for UAV navigation. Furthermore, ref. [28] introduces a method called fiducial marker-corrected stereo visual-inertial localization (FMC-SVIL), designed to operate on a limited-resource onboard computer. This method aims to accurately determine the global position of a UAV beneath bridge girders. The benefits of stereo vision camera configurations in autonomous UAV navigation can be found in [29]. Moreover, ref. [30] introduces an alternative method for reducing the cumulative error in each individual frame. Specifically, a fundamental issue with the pure VO method is addressed: why relatively large errors are occasionally produced by the camera pose estimation process, even when the residual of reprojection error (RPE) is well controlled. Experiments were conducted using UP Board onboard computers for indoor UAV navigation. Results from benchmark and onboard tests showed that the proposed method’s performance is comparable to leading stereo or visual-inertial odometry methods in terms of accuracy and robustness.

The problem of detecting and avoiding obstacles using computer vision has been investigated in many references. The authors of [31] present an avoidance trajectory planning algorithm that was developed for single-camera-based Sense and Avoid (SAA) applications. This study targets two primary challenges: obstacle localization based on monocular vision and planning collision avoidance trajectories around obstacles. The solution was developed, integrated, and validated by inducing collisions between two UAVs through various experimental flight tests in [32]. Furthermore, ref. [33] investigates a novel collision avoidance system utilizing a monocular camera and an intelligent algorithm for real-time obstacle avoidance processing, while [34] presents a mathematical model and an algorithm for measuring the depth of a UAV with any static frontal obstacle. In [35] a deep reinforcement learning approach is presented for vision-based navigation of UAVs in avoiding stationary and mobile obstacles. The research aims to use reinforcement learning algorithms to autonomously train a drone to navigate around obstacles, utilizing both discrete and continuous action spaces, solely relying on image data. Alternatively, the landing phase poses a significant challenge for the UAV, with the precision and success rate of autonomous landing often determining the overall success of the mission. A review of vision-based autonomous landing for UAVs can be found in [36]. While [37] proposes a UAV vision-aided INS/Odometer system for land vehicle autonomous navigation, the authors of [38] investigate a UAV safe landing navigation process that depends on streamlined computer vision modules capable of running efficiently on the constrained computational resources available on a typical UAV. While current methods have successfully addressed the issue of accurate navigation and landing with the UAV’s built-in visual system, most of these studies are limited to daytime or well-lit laboratory settings. Thus, ref. [39] proposes a system that effectively improves the quality of images captured onboard, ensuring reliable detection and validation of landing markers. This is achieved through a hierarchical decision tree approach, which extracts crucial information.

Compared to prior studies, this research presents a novel approach to detecting and localizing UAVs in indoor environments. The proposed system introduces the following innovations:

- Implementation of a cost-effective indoor positioning system utilizing basic cameras, eliminating the necessity for optical cameras or tracking systems.
- Utilization of a computer vision approach (specifically, the YOLO architecture) for UAV detection.
- Creation of a new manually labeled dataset specifically tailored for multi-rotor UAVs.
- Experimental validation of the system’s performance.

## 3. Hardware and Materials

### 3.1. System Architecture

**Figure 1:**
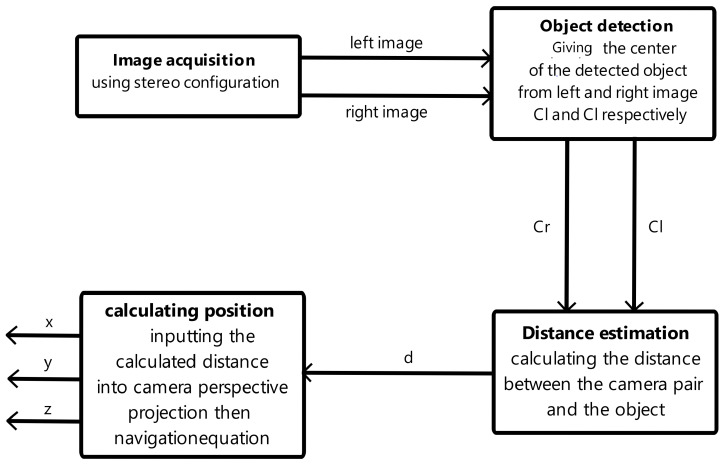
IPS architecture.

In order to eliminate the dependence on GPS for drone position input, an indoor positioning system was developed, employing a pair of cameras and computer vision techniques for object detection and depth estimation. This innovative system allows the drone’s position to be calculated within the lab environment in real-time, utilizing a customized frame of reference. Figure 1 depicts a comprehensive diagram illustrating the algorithm driving the IPS, highlighting various blocks and demonstrating their interactions.

**Figure 2:**
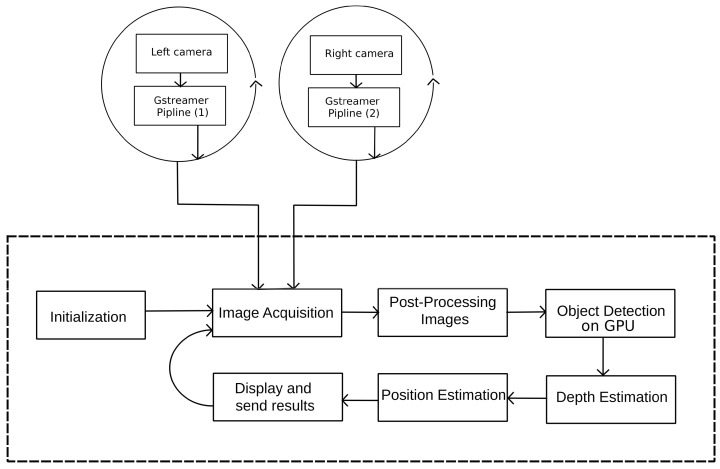
IPS data flow diagram.

Figure 2 illustrates how the proposed system calculates the drone’s position by simultaneously utilizing visual inputs from both cameras. This process begins by defining the operational workspace and aligning it with the overlapping field of view captured by both cameras. For an object to be accurately detected, it must be located within this defined area, ensuring visibility to both the left and right cameras. The stereo setup entails positioning the cameras at a predetermined distance from each other, referred to as the baseline. This separation causes the views from the two cameras to be horizontally shifted.

**Figure 3:**
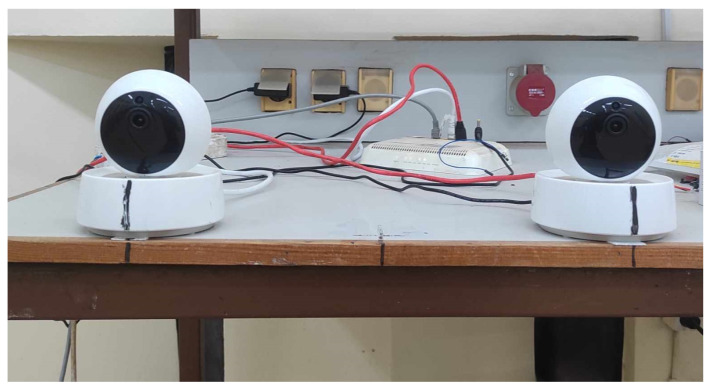
Stereo camera pair.

After configuring the camera vision, it is crucial to meticulously align the optical axes of the cameras to ensure they are both parallel and perpendicular to the baseline with utmost precision (see Figure 3). This step is of significant importance, as even the slightest deviation can greatly impact overall precision. This meticulous alignment is necessary because the system relies on receiving two images with identical orientations. Any inaccuracies in the cameras’ orientation would consequently lead to unmatched images, resulting in errors.

Once the configuration is complete, a connection is established between the cameras and a computer to acquire input images, enabling the reception of all data in synchronized timing. On the software front, multiprocessing is employed by assigning each camera stream to a dedicated thread. This approach allows both data acquisition processes to occur concurrently. Subsequently, each captured image is input into an object detection model. Specifically, YOLOv5 is chosen for its exceptional balance between detection precision and speed. Through the detection generated by both the left and right images, the pixel coordinates representing the center of each detected object are extracted. These pixel coordinates play a crucial role in the forthcoming explanation of the depth estimation technique within this chapter, which serves as the fundamental pillar of our indoor positioning system. Once the distance between the detected object and the camera pair is obtained.

### 3.2. System Configuration

The construction of this system commenced with the acquisition of two identical low-cost WiFi IP cameras. Our selection criteria were based on their favorable resolution (1920 × 1080) and field of view (FOV). Furthermore, these cameras were chosen for their compatibility, as they utilize the RTSP protocol for video output and maintain an acceptable frame rate of 12 fps. However, we encountered an issue with latency at the outset, with approximately a 2 second delay, which contradicted the intended real-time estimation requirement. To measure this latency, we placed the camera’s view on a laptop screen beside a stopwatch and captured the laptop screen to determine the actual stopwatch time and the corresponding image from the camera. This revealed a noticeable 2 second delay between the laptop’s stopwatch and the video stream.

To mitigate this undesired delay, we implemented both hardware and software measures. On the hardware side, we established a connection with the cameras using an Ethernet cable and a hub, reducing latency and enhancing connection stability by minimizing noise. On the software side, we integrated a specialized streaming library called Gstreamer, which facilitated the creation of a pipeline for each camera, enabling concurrent operation. Gstreamer optimized both the latency and quality of the video streams, requiring only the specification of the camera IP while automatically handling the rest of the optimization process. This reduction brought the latency down to nearly 100 ms, deemed suitable for our application.

With images from both cameras obtained with acceptable latency and stability, we turned to the OpenCV library, which offers a wide range of computer vision tools and algorithms to simplify image manipulation. Importantly, OpenCV is compatible with Gstreamer. Following this, we designated the center of the lab as the origin and configured the cameras in a stereo setup, maintaining a baseline of 36 cm between them. These cameras were positioned 3.5 m apart from the origin on a table with a height of 0.89 m, with the right camera aligned with the origin axis.

### 3.3. Images Acquisition and Processing

Our subsequent phase involved establishing connections between each camera and the program using the RTSP protocol via Gstreamer. This setup ensured the simultaneous reception of frames from both the left and right camera streams. To optimize resource utilization, each stream was allocated to a separate processing thread. These threads operated independently from the main program loop, continuously providing frames available in the pipeline at any given moment. This design not only protected the streams’ latency against potential delays or errors in the main program but also shielded them from disruptions that could affect their functionality or cause them to shut down, enhancing the reliability and robustness of our image acquisition process.

Upon acquiring the images, our focus shifted to correcting the distortion effect. This was accomplished using the expression derived during the discussion of distortion effects, along with the correction matrices previously calculated. Subsequently, the images were resized to a resolution of 640 × 640, aligning with the specifications of the YOLOv5 model upon which our system was built. All of these adjustments were implemented using the OpenCV library. This post-processing stage was instrumental in preparing the images for further analysis and detection using our chosen model.

## 4. Drones Detection

### 4.1. Dataset Preparation

Drones integrated with advanced indoor positioning systems offer unmatched performance and versatility. The Pixhawk Drone, equipped with the PIX Hawk autopilot system, delivers exceptional stability and autonomy, enabling precise indoor flight with intuitive controls and real-time telemetry data. Similarly, the Naza Drone, leveraging the renowned Naza M-Lite autopilot system, excels in indoor navigation with intelligent flight modes and GPS-based functions. Moreover, the Parrot Mambo, when paired with the optional FPV camera, seamlessly interfaces with our indoor positioning system, making it an optimal choice for indoor development. With its automatic stabilization system and flexible connectivity options via Wi-Fi and Bluetooth using the PyParrot library with Python, the Parrot Mambo offers unparalleled adaptability and ease of integration for indoor applications.

**Figure 4:**
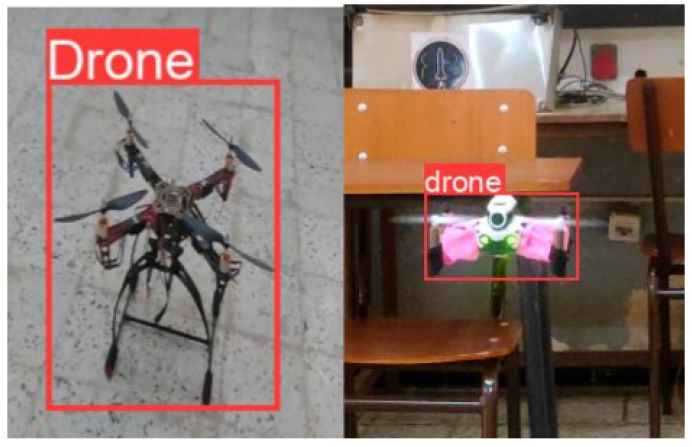
Examples of labeled images.

As indicated in Figure 4, images of the targeted drone were collected, capturing them from various angles and distances. These images underwent meticulous labeling, with corresponding bounding boxes accurately marking the presence of the drone within each image. Subsequently, data augmentation techniques were employed to enhance the diversity and robustness of the dataset. By applying rotations and flips (both horizontal and vertical) to the collected images, the dataset size was significantly increased, resulting in a total of 12,000 labeled images. To prepare for the training process, the dataset was partitioned into three subsets: 85% for training, 10% for validation, and 5% for testing. This allocation, combined with the substantial number of images, proved sufficient for the intended purposes. Given the single-drone scenario and the consistent environment, this setup and dataset size were deemed adequate to achieve accurate detection results.

### 4.2. Model Training

Among the numerous object detection models available, our selection of YOLOv5 is driven by its exceptional balance between performance and precision. This framework offers a remarkable equilibrium that aligns perfectly with our requirements. Notably, YOLOv5’s seamless integration with OpenCV enhances its compatibility and usability within our workflow. A key factor driving our selection is YOLOv5’s foundation on Ultralytics. This platform equips YOLOv5 with an intuitive and user-friendly interface for both training and evaluation, ensuring a clear and straightforward process for harnessing the model’s capabilities.

YOLOv5 primarily uses the Stochastic Gradient Descent (SGD) with momentum as its optimization algorithm. This approach accelerates training by approximating gradients using mini-batches and enhancing convergence stability through momentum, which accumulates a velocity vector in the direction of persistent reduction in the loss function. Additionally, YOLOv5 employs learning rate schedulers, such as warmup and cosine annealing, to adjust the learning rate dynamically during training. These techniques start with a low learning rate that gradually increases, then decrease it following a cosine function to ensure smooth convergence. Together, these methods enhance training efficiency and stability, making YOLOv5 effective for large-scale object detection tasks. The key hyperparameters used for the proposed model are described in Table 1.

**Figure 5:**
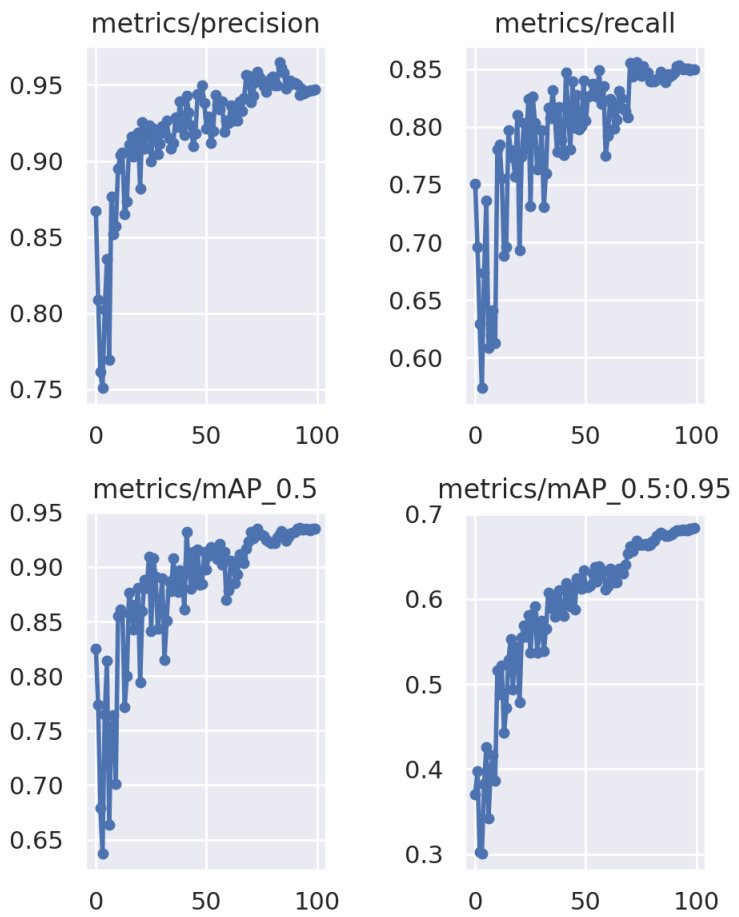
Training results.

The results depicted in Figure 5 demonstrate the success of our training process. After 100 epochs of training, a commendable precision of around 0.9 was achieved by the model. This level of precision surpasses the requirements of our application, reaffirming the effectiveness of our model for the intended task.

### 4.3. Drones Detection

Once the images are processed as described, they are individually fed into the YOLOv5 model loaded through OpenCV. This previously trained model conducts object detection on each input image, resulting in the identification of the drone within the image and the generation of a bounding box around the detected drone. From this process, the drone’s center coordinates, height, and width are extracted, with a specific emphasis on the horizontal coordinates. It is important to note that object detection is computationally intensive and can significantly slow down the system, particularly during image processing and calculation phases. To tackle this challenge, a laptop equipped with a powerful graphics card (RTX 3060) was utilized. This graphics card, with its enhanced computational capabilities, along with the CUDA library, enabled the execution of the object detection process on the GPU rather than the CPU (see Table 2). This efficient approach effectively eliminated delays between data acquisition and object detection, ensuring a consistent frame rate.

## 5. Depth Measurement

At the core of our indoor positioning system lies this computer vision technique. The estimation of the distance between the pair of cameras and the detected object serves as the starting point for position estimation. Without this step, achieving accurate positioning would not be feasible. In our approach, a pair of cameras is utilized, set up in a stereo configuration, providing us with binocular vision. This binocular vision enables the utilization of two different methods: disparity and triangulation, which will be further elaborated upon in this section. However, before any techniques are applied, understanding our camera models and their functioning is crucial. In our case, the pinhole camera model is employed. Subsequently, the identification of imperfections and errors in the input images, which shape our perception of the real world, becomes essential. Correcting these issues is imperative for achieving optimal results.

### 5.1. Depth Measurement with Disparity

**Figure 6:**
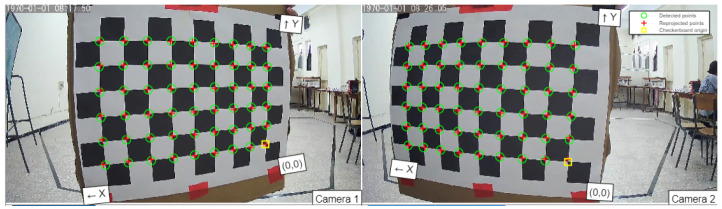
Matlab stereo camera calibrator.

Disparity refers to the difference in horizontal pixel coordinates between corresponding points in two stereo images captured from slightly different viewpoints. When two images of the same scene are taken from different positions, it becomes possible to identify corresponding points in each image that represent the same object or feature in the real world. The horizontal displacement or shift of these corresponding points constitutes the disparity. In most instances of camera calibration, the geometric and optical characteristics of the camera, along with its relative positioning in the World Coordinate System (WCS), are typically determined through a combination of experimentation and computation. Consequently, the calibration process is illustrated in Figure 6.

Within the context of binocular correction, the tasks of distortion elimination and line alignment are performed for both the left and right views. This process is guided by the monocular internal parameters, such as focal length, imaging origin, and distortion coefficients, along with the relative positional relationships, including rotation matrices and translation vectors derived from camera calibration. These adjustments aim to achieve several key outcomes: aligning the imaging origins coordinates for both views, ensuring parallelism of the optical axes of the two cameras, establishing coplanarity of the left and right imaging planes, and aligning epipolar lines. With the aforementioned calibration parameters serving as a foundation, the necessary correction parameters are obtained using the cvStereoRectify function within the OpenCV framework. These acquired parameters are then utilized to rectify the input images for both the left and right perspectives using the cvRemap function. For our application, the obtained parameters are illustrated in Table 3 and Table 4.

In an image, a detected 2D feature represents the perspective projection of a 3D feature in the scene. Several 3D points may project onto the same 2D point, resulting in the loss of depth information. To recover this lost information, two images taken from different perspectives are required. The initial step in recovering 3D spatial information involves establishing the perspective transformation relationship between the scene and its projection onto the left and right images. If a point P, defined by its coordinates (x,y,z) in the real world, projects as the corresponding 2D image coordinates $\left(x_{l},y_{l}\right)$ and $\left(x_{r},y_{r}\right)$ onto the left and right images, respectively, the two cameras are separated by a fixed baseline distance *D* and have a known focal length *f*. Assuming the origin to be at 0, coinciding with the image center in the left camera, the perspective projections can be defined through simple algebra.

(1) $$x=x_{l}\cdot D/d$$

(2) $$y=y_{l}\cdot D/d$$

(3) z=f·D/d

where *d* is defined as the disparity between the two corresponding features in the left and right images:

(4) $$d=|x_{l}-x_{r}|$$

### 5.2. Depth Measurement with Triangulation

**Figure 7:**
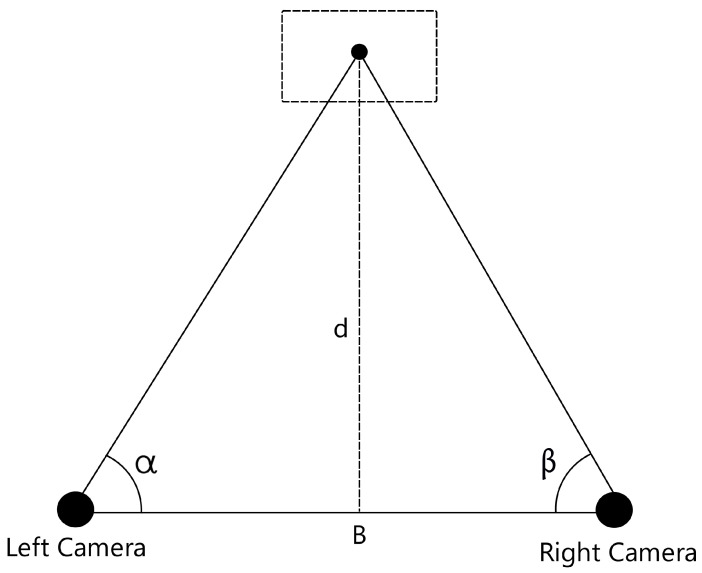
Triangulation representation.

Triangulation, a proficient technique utilized to determine the position of a point, involves measuring the angles to it from two established points situated at the extremities of a fixed baseline. Unlike direct distance measurement in trilateration, this distinctive approach leverages trigonometric principles to derive the coordinates of the point. Subsequently, this point becomes the pivotal third vertex of a triangle, with one side and two angles already ascertained, as illustrated in Figure 7. The inherent simplicity of this method enables its application, particularly in harnessing the potential of a stereo configuration to shape the requisite triangle. The angles necessary for computation are adeptly extracted through the utilization of computer vision, underscoring the precision and efficacy of this methodology. Widely employed by numerous researchers [40,41] due to its effective blend of affordability and dependability, triangulation relies significantly on the alignment and orientation of the cameras, as these factors directly influence angle estimation, subsequently impacting the entire calculation process. Hence, careful precision during the setup of the configuration is vital to guarantee precise results.

**Figure 8:**
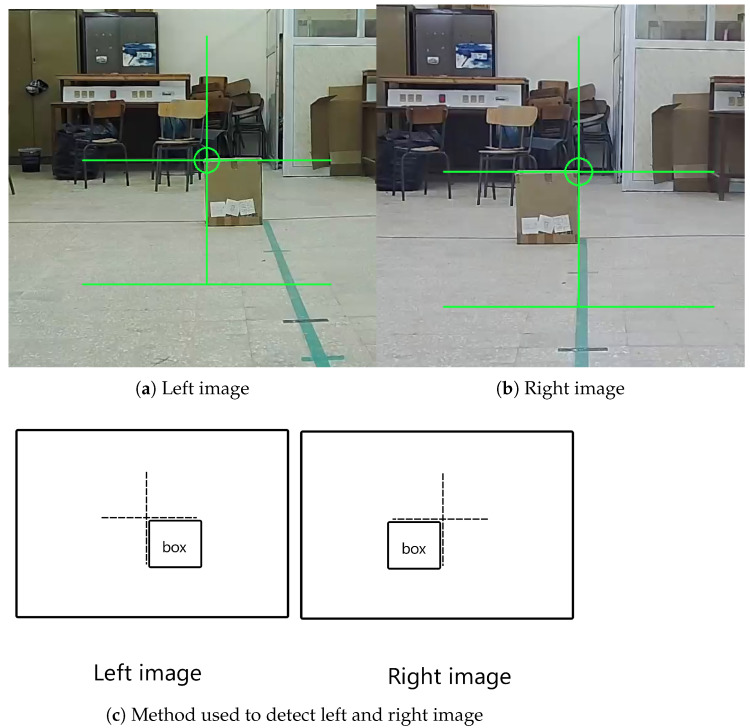
Align cameras using tiered video streams.

First, the cameras’ orientations needed calibration, as detailed earlier. This process involved placing a box with the same width as the baseline behind the origin, positioned 3 m away, marking the extent of our operational space. The box was aligned with the origin axis, resulting in a straight line connecting the origin, the box right side, and the right camera. Subsequently, both camera views were opened on the laptop using OpenCV. Crosshairs were added to the center of each camera’s view, representing the optical axis, aiding in achieving the correct alignment. The primary objective was to ensure that the bottom-right corner of the crosshair in the left image matched the left corner of the box. Similarly, alignment was sought for the bottom-left corner of the crosshair in the right image with the right corner of the box, as illustrated in Figure 8. Precision in this alignment process was paramount, as it directly impacted the overall precision of the system.

The next step involved ensuring that the cameras’ optical axes were perpendicular to the baseline. With the positions of the cameras relative to the lab origin and their orientations fixed, the focus shifted to determining the angle between the cameras’ optical axes and the lab’s horizontal reference plane. This task was simplified by computing a vector orientation that incorporated the right camera. For this purpose, the top-right corner of the box was selected as a second reference point. Consequently, an angle of 4.98° was obtained as a result of this calculation.

Subsequently, the cameras were calibrated by capturing images of chessboard patterns printed on A3 papers with each camera. Utilizing the calibration methods outlined earlier, correction matrices were calculated to address any imaging distortions present. Once the setup was prepared and calibrated, the development of our algorithm commenced. This algorithm played a crucial role in coordinating the cameras and the drone, processing visual data effectively, and advancing our project. Python was exclusively utilized to code the program on a Linux system, as it is highly recommended for computer vision applications. Python, along with C++, was preferred due to its simplicity, performance, and compatibility with the necessary libraries.

**Figure 9:**
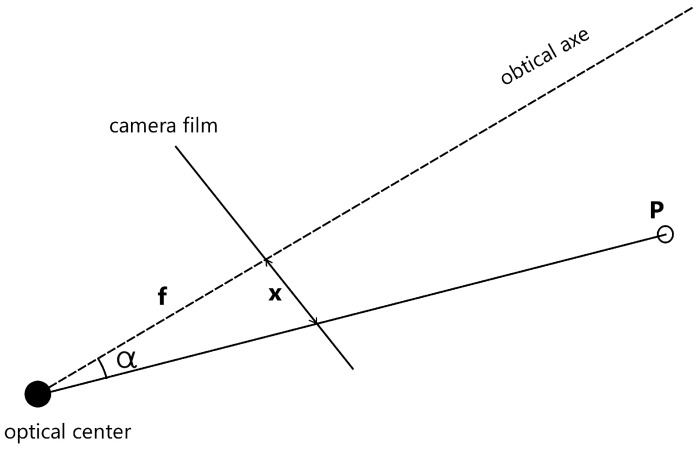
Estimated angles with camera.

For determining the angle of the target object from each camera, the technique discussed in [42] can be employed. This method relies on an understanding of the camera model, basic geometry, and the object’s coordinates within the camera’s film. By utilizing the triangle formed by the optical axis, the detected object, and the camera film, as illustrated in Figure 9, this relationship can be expressed in Equation (5), where f represents the focal length and x denotes the object’s horizontal coordinate in the film reference.

(5) α=arctan(x/f)

Once we have acquired the angle of the object from each camera, the next step involves estimating the distance between the camera pair and the target object. This estimation can be achieved through the utilization of the triangulation (Equation (7)).

(6) $$l=\frac{d}{\tan(\alpha )}+\frac{d}{\tan(\beta )}=d\left(\frac{\cos(\alpha )}{\sin(\alpha )}+\frac{\cos(\beta )}{\sin(\beta )}\right)=d\frac{\sin(\alpha +\beta )}{\sin(\alpha )\sin(\beta )}$$

(7) $$d=l\frac{\sin(\alpha )\sin(\beta )}{\sin(\alpha +\beta )}$$

### 5.3. Depth Estimation Stability

Equipped with the coordinates of the detected object centers, they are directed to one of the algorithms discussed previously. This algorithm undertakes the crucial task of estimating the distance between the object and the drone. It should be noted that the calculated distance is variable, subject to fluctuations due to the inherent instability of detection, especially when one or both of the images fail to detect the object. Even when the object’s position is maintained, slight variations in the detection outcome can still occur. These variations significantly impact the accuracy of depth estimation. To address this challenge, an alpha filter is introduced to the obtained centers. This filter utilizes an initial value derived from the object’s position when it is located at the origin. A threshold of 0.09 is applied to the alpha filter. Mathematically, the process can be represented in Equation (8):

(8) $$\begin{array}{c} C_{L_{n}}=0.09C_{L_{n}}+\left(1-0.09\right)C_{Ln-1}\\ C_{R_{n}}=0.09C_{R_{n}}+\left(1-0.09\right)C_{Rn-1} \end{array}$$

where $C_{R}$ and $C_{L}$ are the horizontal center of the detected object from the right and the left image, respectively.

Through the implementation of this approach, the stability of our depth estimation is enhanced. This accounts for the inherent fluctuations in the detection process and contributes to more consistent and reliable depth calculations.

## 6. Position Estimation

Proceeding with the calculation of the object’s coordinates involves performing backward camera projection, as elucidated in [43]. This is achieved through the translation of object coordinates from the image frame to the world frame. The pixel coordinates denote the position from the top-left corner of the camera’s film to the desired pixel within it, depicted by the vectors (u,v). Conversely, the coordinates in the film reference indicate the position of the desired pixel on the camera’s film plane, originating from the intersection point of the optical axis, denoted by (x,u). A resolution of 1920 × 1080 is utilized for this purpose. The film coordinates can be calculated using the following equations:

(9) x=u−(W/2)

(10) y=(H/2)−v

The transition from film reference to camera reference, representing the position of a given point in space with the camera’s optical axis as a reference, is achieved by applying basic perspective projection using the following equations:

(11) X=x·Z/f

(12) Y=y·Z/f

where *Z* is the calculated distance between the camera pair and the detected object, while *O* is the optical center, *f* is the focal length distance between the camera’s film and the sensor, (x,y) film coordinates and X,Y,Z are camera coordinates.

In our application, the room serves as our main reference point. The conversion of camera coordinates to real-world coordinates begins with the measurement of the exact world position of the chosen right camera $P_{wc}\left(U_{c},V_{c},W_{c}\right)$, which serves as our reference for the conversion process. To simplify this, the right camera is aligned with the room’s horizontal axis, with $U_{c}$ set to zero. The height of the camera from the floor determines $V_{c}$, while its distance from the origin *O* determines $W_{c}$. Additionally, the angle between the camera pair and the floor θ is calculated for later use in rotations.

Advancement is made by aligning the camera frame axes with the world frame axes. This is achieved through the sequential application of two rotation matrices: first along the X-axis by an angle of θ, followed by a rotation along the Y-axis by an angle of $180^{\circ }$. The resulting combined rotation matrix *R* represents the final orientation.

(13) $$R_{X}=\left(\begin{array}{ccc} 1 & 0 & 0\\ 0 & \cos(\theta ) & \sin(\theta )\\ 0 & -\sin(\theta )/ & \cos(\theta ) \end{array}\right)$$

(14) $$R_{Y}=\left(\begin{array}{ccc} \cos(180^{\circ }) & \sin(180^{\circ }) & 0\\ -\sin(180^{\circ }) & \cos(180^{\circ }) & 0\\ 0 & 0 & 0 \end{array}\right)$$

(15) $$R=R_{Y}R_{X}$$

In [43], it is explained that the transition from the world coordinate system to the camera coordinate system is achieved using Equation (16). Conversely, by inverting Equation (17), the transformation from the camera coordinate system back to the world reference can be established, as detailed in Equation (18).

(16) $$P_{world}=R\left(P_{camera}-Pwc\right)$$

(17) $$P_{camera}=R^{-1}P_{camera}+Pwc$$

(18) $$\left(\begin{array}{c} U\\ V\\ W\\ 1 \end{array}\right)=\left(\begin{array}{cccc} R_{1,1} & R_{2,1} & R_{3,1} & 0\\ R_{1,2} & R_{2,2} & R_{3,2} & 0\\ R_{1,3} & R_{2,3} & R_{3,3} & 0\\ 0 & 0 & 0 & 1 \end{array}\right)\left(\begin{array}{c} X\\ Y\\ Z\\ 1 \end{array}\right)+\left(\begin{array}{c} Uc\\ Vc\\ Wc\\ 0 \end{array}\right)$$

Ultimately, the results are showcased on the right camera’s stream, which was selected as our reference in this approach. This involves drawing a bounding box around the detected drone and supplementing it with the corresponding world coordinates. This visual representation offers a clear and immediate understanding of the drone’s position within the environment. Finally, as the estimated position is ascertained, our system fulfills its primary purpose by wirelessly transmitting this vital information to the drone within the indoor environment. This step is pivotal, enabling the drone to navigate adeptly within the indoor space, guided by real-time feedback from our system.

## 7. System Evaluation

**Figure 10:**
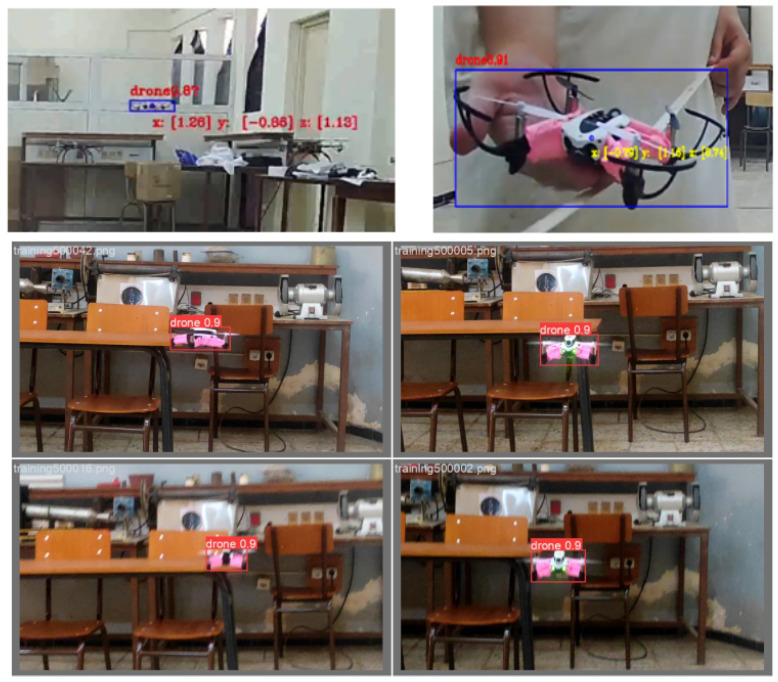
Captures of IPS outputs.

In order to comprehensively evaluate the effectiveness and performance of the developed indoor positioning system, a meticulous evaluation process was executed. This assessment primarily focused on core variables and estimation sources, particularly the coordinates derived from the cameras and the depth estimation methodology used to determine the distance between camera pairs and detected objects. The accuracy of the system’s coordinates remains contingent upon the efficacy of the depth estimation method employed, as the camera pair configuration remains unchanged. An illustration of the obtained results can be found in Figure 10. A video demonstration showcasing the system in action is available at: https://youtu.be/dWLj9sYYK4w (accessed on 9 May 2024).

To establish quantifiable measures for comparison against the estimated values, measurements of distances between the center of the baseline and randomly selected points across the operational space were conducted. This measurement span ranged from 0.5 m to 5 m, accomplished using a tape measure. With these measured distances in hand, the positions within the laboratory frame were then calculated. Using the series of translation steps observed previously, the measured distances were mapped onto the lab’s spatial coordinates. This process enabled a comprehensive evaluation of the system’s accuracy and alignment with real-world distances across the designated operational range, yielding the following values in Table 5.

**Figure 11:**
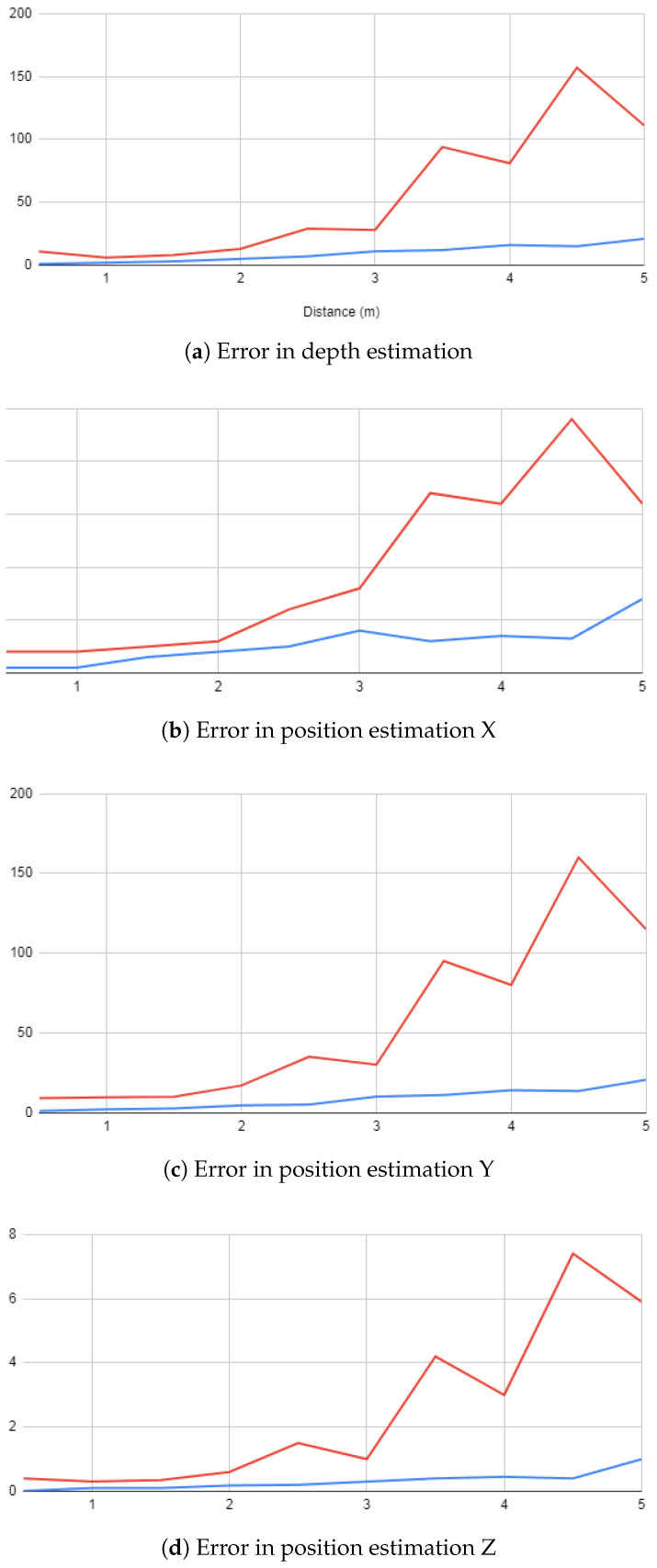
Error estimation in (**a**) depth (**b**) X (**c**) Y (**d**) Z.

**Figure 12:**
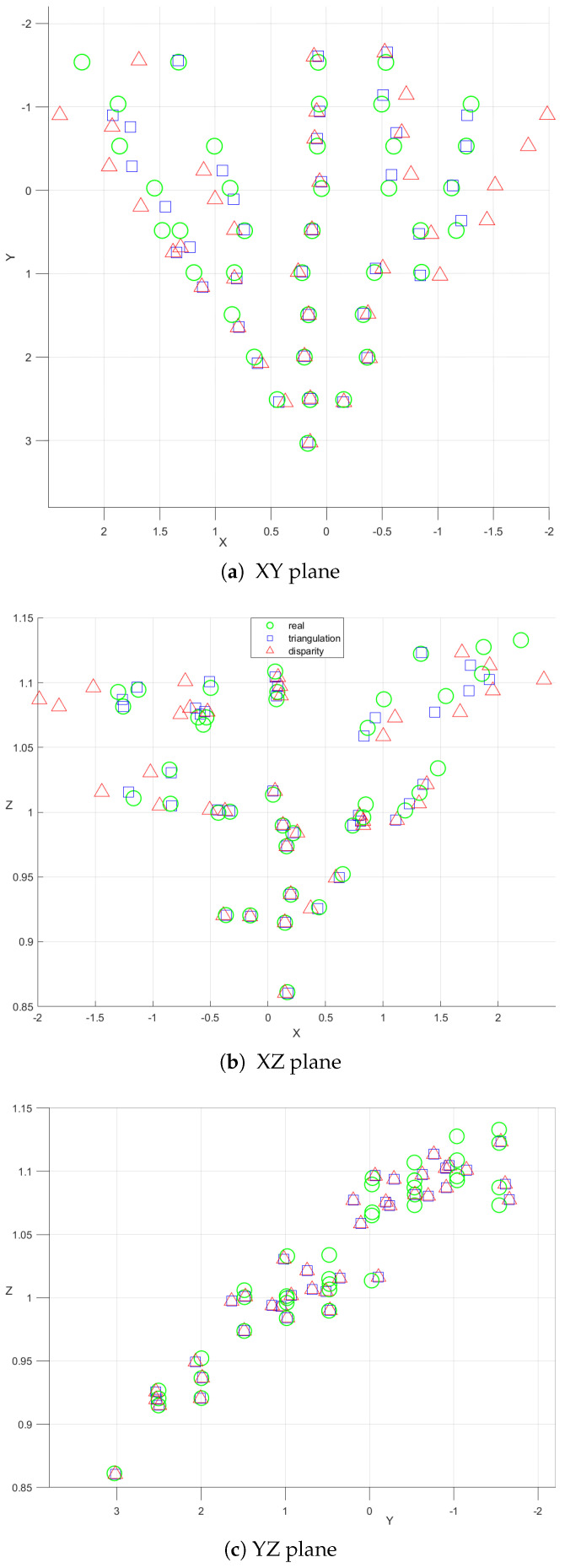
Position estimation on (**a**) XY (**b**) XZ (**c**) YZ planes.

To facilitate a comprehensive understanding and to clearly differentiate between the two methods employed in our study, our discussion is supplemented with a series of experiments illustrated in Figure 11 and Figure 12. These visual aids serve as valuable tools that help to define the subtle differences and variations between the methods in a more easily understandable way.

The results obtained from our comprehensive evaluation provide promising insights into the potential of the developed system. This system, tailored for indoor navigation and position estimation, emerges as a valuable and cost-effective solution. Particularly, the results underscore the viability of the triangulation method over the disparity method. This distinction arises from the disparity method’s increased reliance on the stereo configuration and the overall performance of the employed cameras. Given that our system simulates a stereo camera setup, it becomes evident that adopting a prebuilt stereo camera with established calibration would significantly enhance the effectiveness of the disparity approach.

In contrast, the efficacy of the triangulation method hinges on the accurate estimation of angles from each camera’s viewpoint and the precision of object detection. These factors collectively contribute to mitigating errors in the positioning process. Additionally, our analysis reveals a common trend in both methods, wherein precision diminishes with an increasing distance between the detected object and the camera pair. This trend stems from a fundamental principle inherent in stereo configurations: as an object moves farther away from the cameras, the corresponding disparity decreases. Consequently, this reduction in disparity can hinder the system’s ability to accurately detect changes in the object’s position or orientation, particularly as the object moves further from the camera pair.

Table 6 provides a comprehensive overview of three different indoor positioning technologies: the proposed system utilizing Computer Vision, RFID Positioning, and Wi-Fi Positioning. Each technology is evaluated across various criteria including precision, real-time processing capabilities, scalability, non-intrusiveness, environmental impact, cost, and deployment complexity. The proposed Computer Vision system demonstrates high precision by leveraging high-resolution camera data, ensuring accurate indoor localization. It also offers real-time processing capabilities with minimal infrastructure changes and low deployment complexity, making it an attractive option for indoor positioning applications. In contrast, RFID Positioning relies on tags and readers, leading to higher costs and deployment complexity, while Wi-Fi Positioning is susceptible to signal interference and environmental obstacles. This comparison highlights the strengths and weaknesses of each technology, aiding decision-making for indoor positioning system selection based on specific requirements and constraints.

## 8. Conclusions

In conclusion, the development and evaluation of the indoor positioning system have provided invaluable insights into its performance and potential applications. While the current iteration of the system shows promising outcomes, there are opportunities for refinement and advancement. Significant potential exists for substantial enhancements in both performance and precision, which could be realized through the optimization of hardware, the integration of supplementary position estimation sources for heightened redundancy, and the refinement of the algorithmic framework. The implications of this work extend beyond indoor positioning, potentially benefiting various domains such as robotics, navigation, and augmented reality applications. The successful combination of advanced measurement techniques, translation processes, and depth estimation methods underscores the potential impact of the system on addressing real-world positioning challenges.

In essence, this endeavor marks a significant stride toward achieving reliable indoor positioning. As technology continues to evolve, the insights gained from this work provide a solid foundation for further advancements in spatial estimation and its diverse applications. Future works could explore novel sensor fusion techniques, leveraging emerging technologies like IMU and LiDAR, to further enhance accuracy and robustness. Additionally, incorporating machine learning algorithms for real-time adaptation and scene understanding could lead to more intelligent and adaptable indoor positioning systems. Moreover, investigating the integration of indoor positioning with context-aware applications, such as personalized navigation and location-based services, could unlock new possibilities for enhancing user experiences in indoor environments.

## Figures and Tables

**Table 1:** Key YOLOv5 hyperparameters.

Hyperparameter	Default Value	Description
lr0	0.01	Initial learning rate
momentum	0.937	SGD momentum
weight_decay	0.0005	L2 weight decay
warmup_epochs	3.0	Number of warmup epochs
box	0.05	Box loss gain
cls	0.5	Classification loss gain
obj	1.0	Objectness loss gain
iou_t	0.20	IoU training threshold
hsv_h	0.015	HSV-Hue augmentation
hsv_s	0.7	HSV-Saturation augmentation
hsv_v	0.4	HSV-Value augmentation
fliplr	0.5	Image flip left-right (probability)
mosaic	1.0	Mosaic augmentation (probability)

**Table 2:** System environment.

OS	Ram	Graphics Card	Prossecer	Python	Editor
Ubuntu 20.04	32gb ddr4	nvdia rtx 3060	ryzen 5600h	version 3.8	pycharm

**Table 3:** Left and right camera intrinsic parameters.

Property	Value	
	Left	Right
**Focal length**	[1418, 1416.3]	[1421.8, 1419.8]
**Principal Point**	[1024.9, 592.2045]	[1038.5, 586.2937]
**Image size**	[1080, 1920]	[1080, 1920]
**Radial Distortion**	[−0.4068, 0.1560]	[−0.4029, 1.1481]
**Tangential Distortion**	[0, 0]	[0, 0]
**Skew**	0	0

**Table 4:** General camera intrinsic parameters.

Property	Value
**Dimensionality**	3
**R**	$\left[\begin{array}{ccc} 1 & -0.0086 & 0.005\\ 0.0087 & 0.9999 & -0.0128\\ -0.0049 & 0.0129 & 0.9999 \end{array}\right]$
**Translation**	[−285.1561, 1.7666, 27.1272]

**Table 5:** Average errors for depth estimation.

Distance (m)	Δe with Triangulation (cm)	Δe with Disparity (cm)
0.5	1	11
1	2	6
1.5	3	8
2	5	13
2.5	7	29
3	11	28
3.5	12	94
4	16	81
4.5	15	157
5	21	111

**Table 6:** Comparison of indoor positioning systems.

Criteria	Proposed System (Computer Vision)	RFID Positioning	Wi-Fi Positioning
Precision	High (utilizes high-resolution camera data)	Moderate (depends on tag-reader distance)	Moderate (affected by signal strength variations)
Real-Time Processing	Yes	Limited (depends on tag scanning rate)	Limited (depends on signal processing speed)
Scalability	High (minimal infrastructure changes)	Moderate (requires deployment of tags and readers)	Moderate (requires signal access points and calibration)
Non-Intrusiveness	Yes (no additional hardware on UAVs)	No (requires tags on UAVs)	Yes (no additional hardware on UAVs)
Environmental Impact	Low (less affected by obstacles)	High (signal interference from obstacles)	High (signal interference from walls and objects)
Cost	Moderate (cost of cameras and processing units)	High (cost of tags and readers)	Moderate (cost of access points and calibration)
Deployment Complexity	Low (simple camera setup)	High (extensive tagging and reader setup)	Moderate (requires access point placement and calibration)

## Data Availability

Dataset available on request from the authors.
